# Off-target effects dominate a large-scale RNAi screen for modulators of the TGF-β pathway and reveal microRNA regulation of *TGFBR2*

**DOI:** 10.1186/1758-907X-2-3

**Published:** 2011-03-14

**Authors:** Nikolaus Schultz, Dina R Marenstein, Dino A De Angelis, Wei-Qing Wang, Sven Nelander, Anders Jacobsen, Debora S Marks, Joan Massagué, Chris Sander

**Affiliations:** 1 Computational Biology Program, Memorial Sloan-Kettering Cancer Center, New York, NY, USA; 2 Cancer Biology and Genetics Program, Memorial Sloan-Kettering Cancer Center, New York, NY, USA; 3 High-Throughput Screening Core Facility, Memorial Sloan-Kettering Cancer Center, New York, NY, USA; 4 Cancer Center Sahlgrenska, University of Gothenburg, Gothenburg, Sweden; 5 Department of Systems Biology, Harvard Medical School, Boston, MA, USA

## Abstract

**Background:**

RNA interference (RNAi) screens have been used to identify novel components of signal-transduction pathways in a variety of organisms. We performed a small interfering (si)RNA screen for novel members of the transforming growth factor (TGF)-β pathway in a human keratinocyte cell line. The TGF-β pathway is integral to mammalian cell proliferation and survival, and aberrant TGF-β responses have been strongly implicated in cancer.

**Results:**

We assayed how strongly single siRNAs targeting each of 6,000 genes affect the nuclear translocation of a green fluorescent protein (GFP)-SMAD2 reporter fusion protein. Surprisingly, we found no novel TGF-β pathway members, but we did find dominant off-target effects. All siRNA hits, whatever their intended direct target, reduced the mRNA levels of two known upstream pathway components, the TGF-β receptors 1 and 2 (*TGFBR1 *and *TGFBR2*), via micro (mi)RNA-like off-target effects. The scale of these off-target effects was remarkable, with at least 1% of the sequences in the unbiased siRNA library having measurable off-target effects on one of these two genes. It seems that relatively minor reductions of message levels via off-target effects can have dominant effects on an assay, if the pathway output is very dose-sensitive to levels of particular pathway components. In search of mechanistic details, we identified multiple miRNA-like sequence characteristics that correlated with the off-target effects. Based on these results, we identified miR-20a, miR-34a and miR-373 as miRNAs that inhibit *TGFBR2 *expression.

**Conclusions:**

Our findings point to potential improvements for miRNA/siRNA target prediction methods, and suggest that the type II TGF-β receptor is regulated by multiple miRNAs. We also conclude that the risk of obtaining misleading results in siRNA screens using large libraries with single-assay readout is substantial. Control and rescue experiments are essential in the interpretation of such screens, and improvements to the methods to reduce or predict RNAi off-target effects would be beneficial.

## Background

RNA interference (RNAi) has emerged as a central tool to analyze the function of mammalian genes, both *in vitro *and *in vivo*. The technology has been widely used in mammalian cells to suppress the expression level of individual genes, thus helping to define the functional roles of genes, particularly in disease. RNAi screens, using either double-stranded RNA in *Drosophila *cells, or small hairpin (sh)RNA or small interfering (si)RNA libraries in mammalian cells, have been used for the identification of novel components of a variety of signal-transduction pathways [1]. Large-scale siRNA screens in mammalian cells have been performed to identify modulators of the cell cycle [2], nuclear factor-κB signaling [3] and β-catenin signaling [4], and also in the study of infectious diseases [5-10] and in stem cells [11,12]. Much work has centered around siRNA design algorithms, with a focus on gene-target specificity and efficiency [13-17]. However, individual siRNAs have been shown to downregulate tens or even hundreds of genes by binding in a micro (mi)RNA-like manner to the 3' untranslated regions (UTRs) of off-target mRNAs [18-22]. Screens attempt in a number of ways to control for these off-target effects [23], but results must still be interpreted cautiously.

Transforming growth factor (TGF)-β is part of a large metazoan family of multifunctional cytokines involved in a wide range of cellular processes, including proliferation, apoptosis, differentiation and migration. Although its functions are diverse, TGF-β is the archetypal anti-mitogenic cytokine, affecting a wide variety of epithelial and endothelial cells, and involving widely divergent cellular processes, such as cytostasis, apoptosis and induction of cellular senescence [24]. As part of a signal-transduction pathway integral to mammalian cell proliferation and survival, aberrant TGF-β responses have been strongly implicated in neoplastic development [25,26]. The loss of the cytostatic response to TGF-β, coupled with retention of TGF-β signaling components, results in the *in vivo *selection of more aggressive tumors [27]. In the absence of the cytostatic program, TGF-β promotes increased invasive capability as a result of induced epithelial-mesenchymal transdifferentiation [28]. TGF-β signaling is mediated by a short cascade: binding of TGF-β to a type II TGF-β receptor (TGFBR2) leads to recruitment and phosphorylation of a type I receptor (TGFBR1), which can then phosphorylate the transcription factors SMAD2 and SMAD3. Phosphorylated SMADs accumulate in the nucleus, where they form complexes with SMAD4 and other transcriptional regulators, and activate or repress the transcription of many target genes [24]. Modulation of cell responsiveness to TGF-β can theoretically occur at any of the several steps of the signaling pathway from the membrane to gene-promoter regions, and mutations of several of the integral pathway components have been characterized in certain cancers [29-31]. However, only the two TGF-β receptors TGFBR1 and TGFBR2 have thus far been shown to be essential, non-redundant pathway components required for SMAD phosphorylation and subsequent nuclear translocation.

To identify novel components and modulators of the TGF-β pathway, we performed a large-scale (6,000-gene) functional RNAi screen for genes affecting TGF-β-induced nuclear translocation of a green fluorescent protein (GFP)-SMAD2 fusion protein in human keratinocytes (HaCaT). We identified and validated 176 siRNAs that negatively regulate GFP-SMAD2 nuclear localization in response to TGF-β. Further analysis of these siRNAs revealed a common mechanism of influence on TGF-β signal transduction through miRNA-like off-target effects on *TGFBR1 *and *TGFBR2*. In particular, *TGFBR2 *was the more frequent target of silencing, and an unexpectedly large number of siRNAs targeted both receptors.

We investigated the relationship between sequence complementarity of the target site, the number of sites and the screen ranking to determine the rules for this miRNA-like targeting. Our results validate rules recently identified by others, and we provide further evidence for the importance of sequence complementarity outside of the siRNA seed region.

## Results

### A 6,000-gene siRNA screen to look for TGF-β pathway components

**Figure 1:**
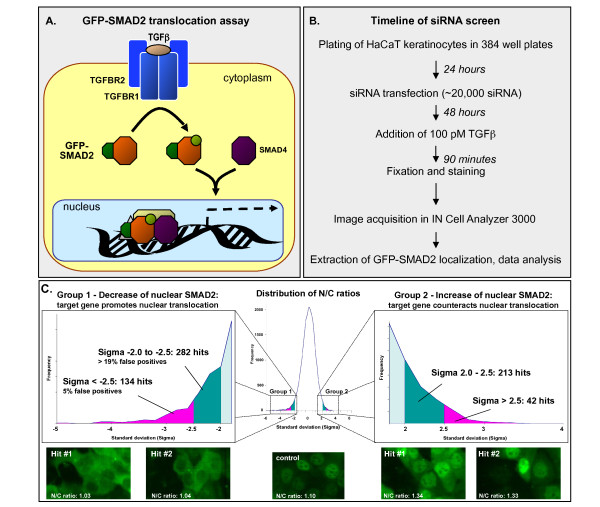
**Results of a 6,000 gene small interfering (si)RNA screen using a green fluorescent protein (GFP)-SMAD2 translocation assay**. **(A) **GFP-SMAD2 translocation assay in the context of the transforming growth factor (TGF)-β pathway. **(B) **Screen timeline. **(C) **Distribution of normalized nuclear:cytosolic (N:C) ratios for all siRNAs used in the screen. Screen 'hits' show increased or decreased GFP-SMAD2 nuclear translocation relative to the mean after TGF-β stimulation. The N:C ratio was determined by image analysis of an average of 220 cells per well. Group 1 comprised 134 small interfering (si)RNAs that led to a decrease in GFP-SMAD2 translocation (σ > 2.5), whereas group 2 comprised 42 siRNAs that led to an increase in GFP-SMAD2 translocation (σ > 2.5).

The screen used a nuclear translocation assay consisting of a GFP-SMAD2 fusion protein stably expressed in HaCaT keratinocytes (Figure 1A) [32]. Translocation of this reporter from the cytoplasm to the nucleus can be tracked and quantified by fluorescence microscopy. Using a 384-well format, we transfected our assay cell line with a 6,000-gene siRNA library consisting of multiple independent siRNAs targeting each gene (total of 21,000 siRNAs) (Figure 1B). Positive (siRNAs targeting TGFBR2) and negative (non-targeting siRNAs) transfection controls were included on every plate. Two days after transfection, cells were stimulated with TGF-β, and the nuclear translocation of SMAD2 was quantified using the mean nuclear:cytosolic (N:C) localization ratio of GFP-SMAD2. This ratio was assessed by automated confocal microscopy using GFP intensities from an average of 220 cells per well. A higher N:C ratio (> 1.1) indicates nuclear localization, whereas a lower N:C ratio (< 1.1) is indicative of a predominantly cytosolic localization. An siRNA causing a significant deviation (positive or negative) of the N:C ratio from the mean is expected to have affected a gene involved in the TGF-β pathway. A change in the N:C ratio can be caused by facilitation or inhibition of TGF-β-induced GFP-SMAD2 phosphorylation or nuclear translocation.

We found a correlation between cell density and N:C ratio, and normalized for this effect (see Methods). This normalization procedure had a large effect on N:C ratios for the siRNAs that caused an increase in N:C ratio, but only a small effect on the siRNAs that caused a decrease in N:C ratio (see Additional File 1 Figure S1; for full screening results, see Additional File 2 Table S1; selected representative images are available at http://cbio.mskcc.org/tgf-beta_screen/).

#### A number of siRNAs caused a significant change in nuclear localization of GFP-SMAD2

Using normalized N:C ratios, we found that 136 siRNAs caused a significant decrease, and 43 a significant increase, in nuclear translocation relative to the mean when stimulated with TGF-β (using a cut-off of 2.5 standard deviations (SDs)) (Figure 1C). The siRNAs that caused an increase in N:C ratio were predominantly affected by large variations in cell density. We performed experimental validation on the four siRNAs that caused the greatest increase, but could not reproduce the effects (data not shown). We therefore concluded that most of these siRNAs were false positives caused by the sensitivity of the assay to changes in cell density, and we focused all subsequent analyses on those siRNAs that caused a decrease in N:C ratio, because these were much less affected by variations in cell density.

#### Effects of screen hits were reproducible

**Figure 2:**
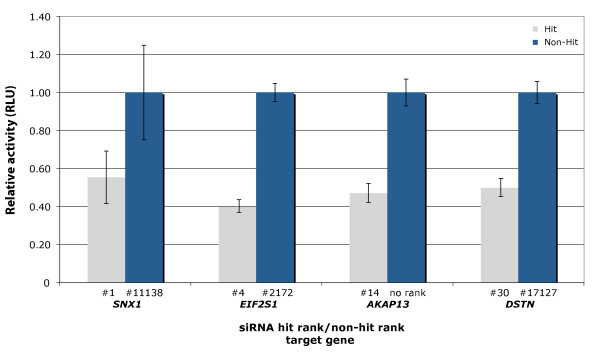
**Effects of small interfering (si)RNA hits on transforming growth factor (TGF)-β signaling validated by various methods**. **(A) **Immunodetection of receptor-phosphorylated SMAD2 in extracts of HaCaT cells transfected with the indicated siRNAs and treated with 100 pmol/l TGF-β for 30 minutes. Numbers represent ranked siRNA hits. **(B) **Effect of siRNA hits on TGF-β-mediated induction of endogenous gene responses. HaCaT cells were incubated with or without 100 pmol/l TGF-β for 3 hours before harvest. Quantitative PCR analysis was used to determine changes of mRNA levels of indicated genes. The mean ± SD of three experiments is shown. Integers (for example, 1, 19) label ranked siRNA hits.

The siRNA hits resulting in the greatest decrease in the N:C ratios of GFP-SMAD2 were tested for effects on translocation of endogenous SMAD2 in response to TGF-β (data not shown). Initial analysis of a subset of high-ranking siRNA hits identified effects on SMAD2 phosphorylation and TGF-β-dependent gene transcription (Figure 2). Transfection of hit siRNA compared with control (LacZ siRNA) resulted in decreased TGF-β-dependent SMAD2 phosphorylation (as determined by immunoblot analysis) and a decrease in nuclear SMAD2 in response to TGF-β treatment (Figure 2A). Total SMAD2 or SMAD3 protein or mRNA levels were unaffected (data not shown). Induction of endogenous TGF-β target genes *p21 *(*CDKN1A*), *SMAD7 *and *PAI-1 *(*SERPINE1*) was decreased in cells transfected with siRNA hits relative to control (Figure 2B). The tested siRNA hits were also effective at reducing mRNA levels of targeted genes (measured by quantitative (q)PCR) (see Additional File 2 Table S2).

#### siRNA target genes were not responsible for the screen phenotype

After validating the effects of selected hit siRNA on TGF-β signaling, we next tried to identify candidate genes for further validation and characterization.

Attempts to group the genes with siRNA hits by function (based on Gene Ontology and signaling pathway data) did not reveal any over-represented biological processes or signaling pathways, nor was there a significant number of genes previously associated with members of the TGF-β pathway in large-scale protein-protein interaction screens [33-35].

Furthermore, of the ~6,000 genes represented by two to five independent siRNAs, there were no multiple siRNA hits for the same gene. One possible explanation for this is that the library had a low knockdown efficiency in HaCaT cells. Tests of eight sets of three siRNAs designed to target a specific gene found that the ratio of potent siRNAs per gene was somewhat variable, with only one potent siRNA for each of the genes in some cases, and several potent siRNAs in others (data not shown). This confirmed the results of tests performed during the design phase of the library (see Methods) [17], and suggested that only one or two out of three siRNAs per gene could be expected to lead to a potent (> 50%) knockdown. Of 24 tested siRNA hits whose targets were expressed in HaCaT cells, the siRNAs identified as hits were effective in reducing the mRNA levels of their intended targets (see Additional File 2 Table S2). However, more than 20% of the siRNA hits were found to be against gene targets not expressed in HaCaT cells (the assay cell line).

The possibility that the siRNA effect was mediated by something other than the intended target gene (for example, through off-target effects on genes involved in the pathway) was supported by two independent observations. Firstly, the effects on TGF-β signal transduction or receptor mRNAs were not seen using independent, validated siRNAs from commercial sources against any of the selected targets. Secondly, specific chemical inhibition (where applicable) of the siRNA target gene protein products had no effect on TGF-β-mediated gene induction (see Additional File 1 Figure S2).

### Off-target effects on TGF-β receptor mRNAs dominate screen hits

#### TGF-β receptor levels were altered by all screen hits

We saw early evidence that the two most likely candidates for off-target effects were the TGF-β receptors, *TGFBR1 *and *TGFBR2*. Transfection of some of the top-ranked siRNA hits resulted in significant reduction in mRNA levels of the known TGF-β pathway components *TGFBR1 *and *TGFBR2 *(Table 1; see Additional File 2 Table S3). This prompted us to systematically test the other siRNA hits for effects on TGF-β receptor mRNA levels using qPCR or a branched DNA assay. We tested the top 134 siRNAs that had caused a translocation effect with an SD σ < 2.5, an additional 44 siRNAs that had an effect with a SD of 2.0<σ < 2.5 and 15 siRNAs that had received positive scores in a previous small validation screen.

**Table 1:** Repression of *TGFBR1 *and *TGFBR2 *mRNAs by the 193 tested screen siRNA^a ^hits^b^.

Off-target mRNAs affected	Number of siRNAs
*TGFBR1 *only	21**^c^**

*TGFBR2 *only	109

Both *TGFBR1 *and *TGFBR2*	42

No off-target effect (false positives)	21

**^a^siRNA = small interfering RNA**.

**^b^**Screen hits were grouped by their effects on mRNA levels of TGFBR1 and TGFBR2, as determined by branched DNA assay (most siRNA) or quantitative (q)PCR (some siRNA).

**^c^**For the 21 siRNAs without off-target effects, the effects on TGF-β signaling were not verifiable by phospho-SMAD2 western blots or by qPCR of characteristic TGF-β target gene mRNAs (false positives) (see Additional File 2 Table S3 for detailed expression data).

Of these 193 tested siRNA hits (remember that all were designed to reduce expression of their respective target genes), 172 caused an off-target reduction (> 25%) of mRNA levels of at least one of the TGF-β receptors: 21 had an effect on *TGFBR1 *only, 109 had an effect on *TGFBR 2 *only, and 42 had effects on both simultaneously to varying degrees (Table 1). For the remaining 21 siRNAs without off-target effects on either *TGFBR1 *or *TGFBR2 *mRNAs, there was no detectable effect on TGF-β signaling as measured by the induction of transcription of selected TGF-β target genes (*SMAD7*, *CDKN1A*, *SERPINE1*) and phosphorylation of SMAD2 (data not shown). These hits are probably a result of noise in the assay system, and we defined these siRNAs without an effect on TGF-β signaling as false positives, giving a rate of 5% false positives for our screen for siRNA with σ > 2.5.

#### Luciferase assay confirmed that the screen siRNA hits act on the TGFBR2 3' UTR

**Figure 3:**
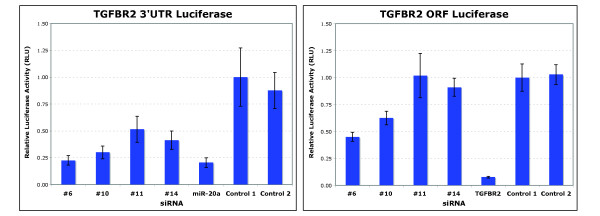
**Confirmation that screen small interfering (si)RNA hits acted primarily on the 3' untranslated region (UTR) of the transforming growth factor-β receptor 2 *(TGFBR2) *mRNA**. Silencing of luciferase-*TGFBR2 *3' UTR or luciferase *TGFBR2 *open reading frame (ORF) protein expression by four representative siRNA hits. Two independent siRNAs were used as non-targeting controls (controls 1 and 2), and a microRNA miR-20a mimic and an siRNA with perfect complementarity to the *TGFBR2 *ORF were used as positive controls for the 3' UTR and the ORF constructs, respectively. The data are reported (*y*-axis) as the relative repression of firefly luciferase expression standardized to *Renilla *luciferase as a transfection control. Integers (for example, 9, 18) label ranked siRNA hits. Error bars on each column are the mean ± SD of three experiments.

Off-target effects have been shown to be mediated by partial complementarity between siRNAs and the 3' UTRs of off-target genes [18-22]. To verify the mechanism of the off-target effects seen in our screen, we tested the effects of selected siRNA hits on the 3' UTR of the *TGFBR2*. We used a luciferase reporter assay in which the mRNA for firefly luciferase was fused to the 3' UTR region or the open reading frame (ORF) for *TGFBR2*, and expressed from a constitutive promoter (Figure 3). Of 13 siRNA hits tested, 11 selectively decreased the activity translated from the luciferase transcript fused to the 3' UTR of *TGFBR2 *(Figure 3). A few siRNAs were also seen to affect the luciferase reporter fused to the ORF, albeit to a much lesser extent. These data confirm the ability of the siRNA hits to mediate the silencing of mRNAs containing the 3' UTR of *TGFBR2*. By contrast, siRNA non-hits (corresponding to the same genes as the siRNA hits) were not able to silence luciferase transcripts containing the 3' UTR of *TGFBR2 *in this system (see Additional File 1 Figure S3).

These results indicate that the HaCaT GFP-SMAD2 translocation system is a useful tool for screening modulators of the TGF-β pathway, as modulation of GFP-SMAD2 translocation correlated well with effects on gene expression of members of the TGF-β pathway, and could be detected and measured with a low rate of false positives. However, interestingly, all siRNAs scoring as hits in the GFP-SMAD2 translocation system had off-target effects on the mRNAs of just two of the known components of the TGF-β pathway. These off-target effects on the receptors immediately explain the effect of these siRNAs in the screen, as SMAD2 is directly downstream of the TGF-β receptors. Thus, contrary to our original intent, we were not able to identify any previously unidentified pathway components using this screen.

### Off-target effects are mediated by miRNA-like target sites

#### Heptamer seed motifs in TGFBR2 are enriched in screen hits

**Figure 4:**
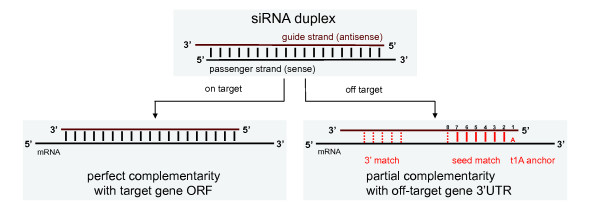
**On-target and off-target mechanisms of small interfering (si)RNAs**. All siRNAs were designed to have perfect complementarity between the siRNA guide strand and the open reading frame (ORF) of the target gene. Off-target effects were mediated primarily through partial complementarity with the 3' untranslated region (UTR) of off-target genes. Complementarity of the siRNA seed (positions 2 to 8), possibly containing G:U wobbles, was thought to be the key determinant Additional known features were the occurrence of an A in the mRNA opposite position 1 of the siRNA (t1A), and partial pairing between the 3' end of the siRNA and the mRNA.

Off-target effects reported in previous RNAi studies were mediated by partial complementarity between siRNAs and the 3' UTRs of off-target genes, involving a heptamer or hexamer 'seed' match of the siRNA strand at the 5' end at positions 2 to 8 or 2 to 7, respectively [18-22]. The mechanism is similar to that of miRNAs, and multiple sequence elements have been proposed to play important roles (Figure 4).

**Figure 5:**
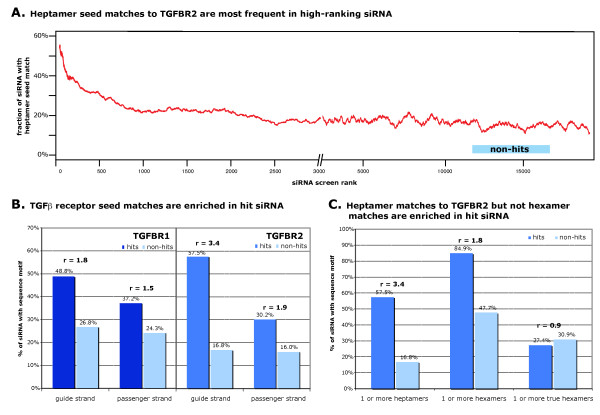
**Sequence signal for off-target effects: small interfering (si)RNA seed matches against the 3' untranslated region (UTR) of transforming growth factor-β receptor 2 *(TGFBR2) *decreased with rank in the screen**. The effects were predominantly mediated by the guide strand, and hexamer seed matches were not effective. **(A) **Moving average of the percentage of siRNA guide strand seed matched to the 3' UTR of the receptors as a function of siRNA rank in the screen (window: 500). **(B) **Off-target effects against transforming growth factor-β receptor 1 *(TGFBR1) *and *TGFBR2 *were mediated predominantly by 3' UTR matches of the siRNA guide strand, but to a small extent by the passenger strand. Enrichment of heptamer seed matches of the guide and passenger siRNA strands to *TGFBR1 *(left) and *TGFBR2 *(right) 3' UTRs are shown for siRNAs with a confirmed effect on *TGFBR1 *and *TGFBR2*. **(C) **Heptamer, not hexamer, seed matches were responsible for *TGFBR2 *silencing. When we analyzed only for the presence of hexamer seed matches that were not part of a heptamer match, there was no enrichment in the *TGFBR2 *hit siRNA compared with control siRNA.

Sequence analysis of all siRNAs used in our screen showed that, compared with siRNA non-hits, a significantly larger percentage of high-ranking siRNAs had heptamer seed matches of the siRNA guide strand against the 3' UTR of *TGFBR2 *(Figure 5A). This percentage dropped rapidly as a function of the screen rank, with a steep decline in the first 200 ranks, followed by a slower decline to a steady level at around rank 2,500. This suggests that there are weak hits in the top 2,500 siRNAs, but the percentage of verifiable hits is much lower than in the top 200. A comparison of siRNA seed sequence matches with the 3' UTRs of all RefSeq genes in the top 200 siRNA screen hits and non-hits identified *TGFBR2 *as the most significantly (off-)targeted gene (see Additional File 3 Table S4), by a wide margin (adjusted p-value = 6.42*10^-12^). A similar analysis using the passenger-strand sequences identified *TGFBR2 *in the top 10 most strongly targeted genes (p-value not significant, see Additional File 3 Table S5), and when sequence data from both strands were combined, TGFBR2 was the only significantly targeted gene (adjusted p-value = 1.22*10^-11^, see Additional File 3 Table S6).

There was no apparent enrichment for seed matches against the *TGFBR1 *3' UTR when the top 200 hits were compared with non-hits. However, we suspected that this enrichment was simply masked by the much larger number of siRNAs targeting *TGFBR2*. Subdividing the verified siRNA hits into *TGFBR1 *and *TGFBR2 *hits based on our mRNA expression data (see Additional File 2 Table S3) revealed an enrichment of seed matches against the *TGFBR1 *3' UTR of the siRNAs that targeted *TGFBR1 *(Figure 5B). For this analysis, only siRNAs causing a knockdown of ≥40% were considered (43 siRNAs with an effect on *TGFBR1 *and 106 with an effect on *TGFBR2*; siRNAs with effects on both receptors were included in both lists). The list of 106 siRNAs with an effect on *TGFBR2 *was used for all further analyses. These 106 siRNAs contained 92 unique seven-mer seed sequences. For the non-hit control group, we used siRNAs ranked between 12,001 and 17,000 (Figure 5A).

The off-target effects we found seem to be overwhelmingly mediated by the guide strand of the siRNA, as suggested by the significant enrichment of guide-strand seed matches against the 3' UTR of *TGFBR1 *and *TGFBR2 *in the siRNA hit group (Figure 5B). However, there was also a smaller but significant enrichment of seed matches in the passenger strand of the siRNA hits, indicating that in some cases the strand designated as the siRNA passenger strand has silencing activity. The preferred incorporation of the guide strand into the RNA-induced silencing complex (RISC) was by design, as the library contains a bias for sequence-directed strand insertion into the RISC [36].

We did not detect enrichment of target sites in the 5' UTR of either *TGFBR1 *or *TGFBR2*. There was only a small enrichment of borderline significance in the ORF of *TGFBR2*, but after removal of sequences with a seed match to the *TGFBR2 *3' UTR, the signal became significant (see Additional File 1 Figure S4).

Both hexamer and heptamer seed matches have previously been attributed to siRNA off-target effects [19]. However, we found that in our data, true hexamer matches (that is, those hexamer matches that were not part of a heptamer match), were not enriched in the hits against *TGFBR2 *compared with non-hits (Figure 5C). We reanalyzed the data by Birmingham *et al. *[19], and noted that the enrichment of hexamer matches reported can be explained exclusively by those hexamer matches that are part of a heptamer match (data not shown). Others have found only a very small contribution of true hexamer seed matches to siRNA off-target effects [37].

The identification of a heptamer sequence base-pairing between siRNAs and target mRNA as part of a mechanism for off-target effects in our screen is supported by several independent studies [18-22,37,38]. However, as with other studies, 'enrichment' of this complementarity motif alone is not enough to explain the hits.

#### Heptamer seed motifs grossly overpredict hits

**Figure 6:**
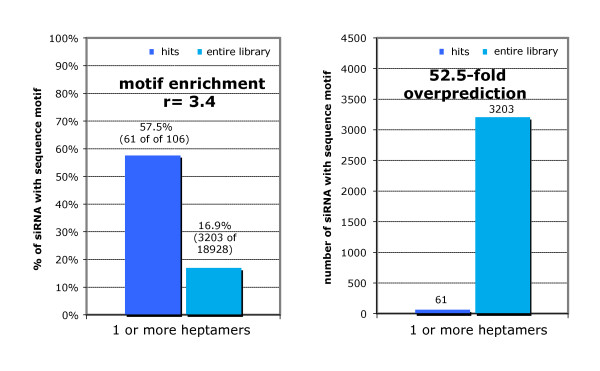
**False prediction of thousands of hits by seed method**. Although we found an enrichment of heptamer seed matches to transforming growth factor-β receptor 2 *(TGFBR2) *in the hit population (left), only 2% of all small interfering (si)RNAs with heptamer seed matched scored as hits (right).

One challenge lies in the understanding why so many of our siRNAs with perfect seed matches to the ORF and 3' UTR of both receptors did not emerge as hits in the assay. Seed-match analysis predicted more than 3,000 of the 20,000 siRNAs in the screen to have potential off-target effects on the 3' UTR of the TGFBR2 mRNA alone, yet only a fraction seemed to be actually effective (Figure 6). Although additional sequence characteristics have been reported [37,38], the price of increased enrichment is typically the identification of many more false negatives. Some groups have attempted to use mRNA secondary structure and site accessibility to aid prediction, with limited success to date [39,40].

Why do siRNAs with identical 'seeds' cause different outcomes in the assay? Evidence suggests three possible reasons: 1) sequence differences outside of the seed that alter their ability to silence the off-target mRNA; 2) different siRNAs have different abilities to be efficiently loaded into the RISC; and 3) assay result variation as a result of technical reasons or biological variability (false negatives). Using sequence analysis, we found a small difference in 3' binding ability (four-mer or five-mer match of the siRNA between positions 10 and 19) between the hit and non-siRNA hits with identical seed sequences. This suggests that differences between these siRNAs can be at least partly explained by sequence differences outside of the seed region. However, experimental testing identified some false negatives with seed heptamers that were identical to those of some siRNA hits. We tested five siRNAs with seed sequences identical to those occurring multiple times within the *TGFBR2 *hits, and found that they caused downregulation of *TGFBR2 *mRNA (qPCR, data not shown).

### Identification of other siRNA (off-)target recognition determinants

In addition to this massive overprediction, more than 40% of our hits did not contain a heptamer motif. Of the 106 verified hits on *TGFBR2*, 61 contained heptamer motifs and 45 did not (Figure 6). Because we are confident that this assay is affected by a drop in either *TGFBR1 *or *TGFBR2 *expression, we wanted to explore additional determinants that are not captured simply by a heptamer match. Theoretically, there are several possible factors: 1) RISC incorporation of the other siRNA strand, 2) effects on the ORF, 3) cooperativity between multiple weaker sites, 4) other match requirements beyond or instead of positions 2 to 8, and 5) accessibility of the site (for example, the two- or three-dimensional structure of the mRNA).

**Figure 7:**
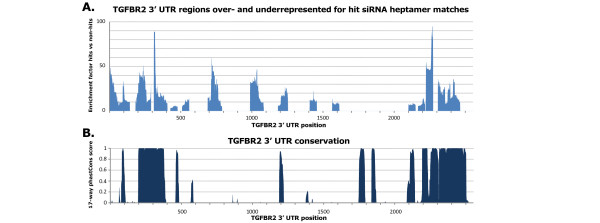
**Conserved regions of the transforming growth factor-β receptor 2 *(TGFBR2) *untranslated region (UTR) were greatly enriched for small interfering (si)RNA hit target sites**. **(A) **Top: Moving average (window size = 50) of the fraction hit siRNA seed sequences with a heptamer match divided by the fraction of non-hit siRNA, showing regions enriched for hit siRNA seed matches along the 3' UTR of *TGFBR2*. **(B) **The vertebrate conservation plot (phastCons 17-way alignment score) shows the correlation between conserved regions and regions enriched for siRNA hits (enrichment factor of 9.8 for heptamer matches in conserved regions).

**Figure 8:**
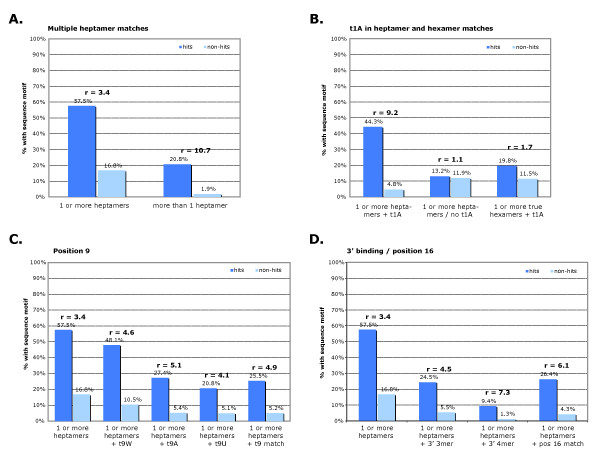
**Importance of various sequence motifs for small interfering (si) RNA off-target effects on transforming growth factor-β receptor 2 *(TGFBR2)***. Percentage of siRNAs with certain sequence motifs found in the *TGFBR2 *3' untranslated region (UTR) are shown for the *TGFBR2 *siRNA hits (dark blue bars) and the non-hit population (light blue bars). Higher blue bars indicate that a prediction based on this feature would be more successful at predicting off-target effects, and the strength of such a pattern is measured by the enrichment factor *r*, which is the ratio of the frequency of a given sequence motif in the seed regions of *TGFBR2 *siRNA hits (dark blue) divided by the frequency of that motif in the seed regions of non-siRNA hits (light blue). **(A) **Multiple heptamers, although rare, were more potent at silencing *TGFBR2*. **(B) **The occurrence of an A in the target *TGFBR2 *UTR opposite position 1 of the siRNA significantly increased the potency of the siRNA (9.2 > 3.4). **(C) **The occurrence of an A or U opposite position 9 of the siRNA or a match at position 9 increased the potency of an siRNA. **(D) **3' pairing, in particular at position 16, seemed to increase siRNA off-target potency. For three-mer and four-mer binding, an offset of no more than two nucleotides was allowed, but the position 16 match was to position 16 of the target.

We used the list of 106 siRNAs with strong effects on *TGFBR2 *to test whether we could confirm previous findings and find additional sequence motifs (Figure 7, Figure 8).

#### Conserved regions of the *TGFBR2 *3' UTR are preferentially targeted by siRNA hits

Heptamer motif analysis of screen hits identified distinct regions of the *TGFBR1 *and *TGFBR2 *3' UTR that seemed to be preferentially targeted by siRNA hits, suggesting the possibility of RNAi 'hotspots' in the 3' UTR (Figure 7). These hotspots overlapped with those regions that are the most highly conserved in vertebrates (Figure 7). Unlike for *TGFBR1*, The targeted regions of *TGFBR2 *were also close to the 5' and 3' ends of the 3' UTR, a phenomenon that has been reported previously for miRNAs and their target genes [38,41,42].

However, we found that although siRNAs with heptamer matches in conserved regions of the TGFBR2 3' UTR (threshold of 0.5, phastCons 17 species) were more likely to silence the TGFBR2 mRNA, only 34 of our verified 106 siRNAs (32% of hits) were now predicted to hit *TGFBR2*. This strongly suggests that conservation should be used as a weighted factor but not as a filter (see Discussion).

#### siRNAs with multiple target sites are more likely to silence *TGFBR2*

We found that siRNAs with multiple heptamer matches against the *TGFBR2 *3' UTR were more likely, by a factor of ~3.0, to silence the *TGFBR2 *mRNA than siRNAs with only one heptamer match (Figure 8A).

#### Target nucleotides opposite siRNA positions 1 and 9 contribute to off-target effects

Our data also reflected a strong prevalence of an adenosine opposite the first position of the siRNA (Figure 8B). This has been termed the 't1A anchor' and previously shown to be an important parameter for miRNA target specificity [43]. In addition, we found a previously described prevalence [37] of A or U at position 9, adjacent to the seed (Figure 8C).

#### Matches of the siRNA 3' end, particularly position 16, increase off-target effects on *TGFBR2*

We analyzed the data for the involvement of base pairing between the 3' half of the siRNA guide strand and the *TGFBR2 *mRNA. Of the siRNAs with heptamer seed matches, we detected enrichment in 3' pairing of three or four bases (between positions 12 and 19 with an offset of no more than two nucleotides) in the *TGFBR2 *siRNA hits compared with siRNA non-hits (Figure 8D). The combination of multiple sequence motifs further increased this enrichment: siRNA hits with all three criteria (a heptamer seed match, a t1A match and with 3' pairing) seemed to be most potent, but only 2.5% of all *TGFBR2 *siRNA hits fulfilled these criteria (Figure 8D).

Performing an analysis of matches in each position revealed a significant enrichment of complementarity at position 16 of the siRNA hits (Figure 8D). These matches tended to occur in the absence of complementary flanking sequence (of the 28 siRNAs with the position 16 match, only eight had an additional flanking match at position 17 or 18, but the 20 matches were isolated). A special significance of position 16 for siRNA targeting efficiency has been reported previously [22], and microarray data of siRNA-transfected cells by Jackson *et al.*, although not discussed in their publication, also showed a correlation between matches at position 16 and off-target effects [20].

We did not observe the position 16 effect in a set of 11 different previously published miRNA transfection experiments in HeLa cells [38,44]. Messenger RNAs with a seed match (seven-mer) in combination with a position 16 match were slightly more repressed after miRNA transfection compared with mRNAs with seed matches alone, but the extent of this effect was not significant (*P *< 0.2, Wilcoxon rank sum test).

#### G:U wobbles in the siRNA 5' end can contribute to off-target effects

**Figure 9:**
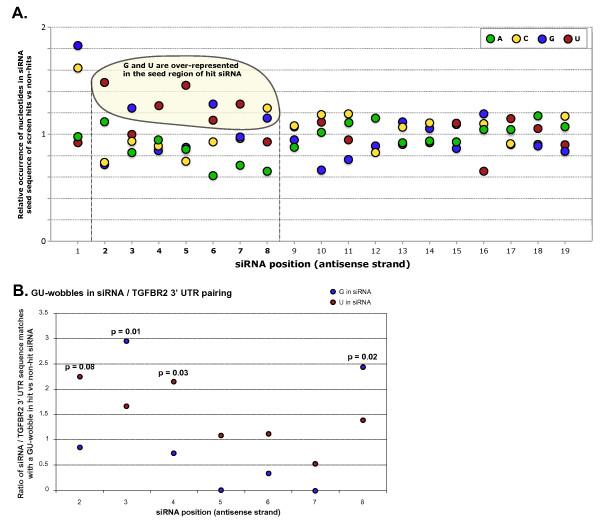
**G:U wobbles in the transforming growth factor-β receptor 2 *(TGFBR2) *3' untranslated region (UTR)/small interfering (si)RNA sequence matches were over-represented in siRNA hits compared with siRNA non-hits, and were position- and strand-specific**. **(A) **Nucleotides observed in individual siRNA positions, shown as the ratio of the frequencies in *TGFBR2 *siRNA hits with unique seeds (n = 92) to frequencies in the entire siRNA library. **(B) ***TGFBR2 *siRNA hits had more *TGFBR2 *3' UTR seed matches containing a G:U wobble at selected positions of the seed region than siRNA non-hits. Enrichments are shown for G and U in individual positions of the siRNA guide strand. *P*-values were calculated by Fisher's exact test.

Imperfect seed matches containing G:U wobbles have been shown to be functional in several examples of miRNAs and their target genes [45,46], but several studies have found G:U wobbles to interfere negatively with miRNA activity [47,48]. G:U wobbles were not previously considered by any of the seed-based miRNA target prediction methods, as the quality of predictions including G:U wobbles was found to be far below that of perfect seed matches [43]. In our results, we found an over-representation of G and U nucleotides in the seed regions of siRNA hits, specifically U bases at positions 2, 4, 5 and 7, and G bases at positions 3 and 6 (Figure 9A), suggesting that some siRNAs may be able to exert their silencing effects through imperfect seed matches containing G:U wobble base pairs.

Allowing for one G:U wobble base-pair of the guide strand with the *TGFBR2 *transcript, with a U at positions 2, 4, 5 or 7 of the siRNA guide strand, we found a small but significant enrichment of seed matches to the *TGFBR2 *3' UTR in the hit versus the non-hit population. The G:U wobbles seemed to occur in specific positions and orientations: positions 2 and 4 (U in the siRNA) and positions 3 and 8 (G in the siRNA) (Figure 9B).

### *TGFBR2 *as a potential miRNA target

The fact that distinct regions of the *TGFBR2 *3' UTR appear to be preferentially targeted by screen siRNA hits suggests the possibility of RNAi 'hotspots' in the 3' UTR, that is, UTR regions particularly accessible to regulation by small RNAs, including expressed miRNAs (Figure 7A). These regions tend to be highly conserved across vertebrate genomes (Figure 7B), and contain predicted target sites for miRNAs. We therefore went beyond the siRNA screen and looked for evidence for repression of *TGFBR2 *by expressed miRNAs (in HaCaT cells), such as miR-20a, miR-93, miR-34a and miR-423, and non-expressed miRNAs, such as miR-373.

**Figure 10:**
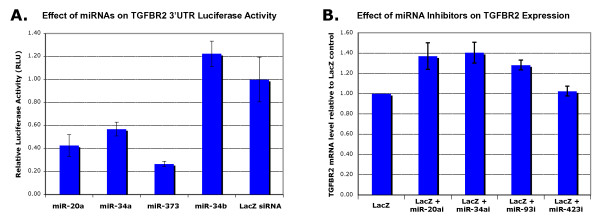
**Regulation of *TGFBR2 *by micro (mi)RNAs**. **(A) **Silencing of luciferase-*TGFBR2 *3' UTR by transfected miRNA mimics. Vertical axis: the relative repression of firefly luciferase expression standardized to *Renilla *luciferase as a transfection control. Error bars = SD for three experiments. miR-20a and miR-34a were expressed in HaCaT cells, whereas miR-373 was not, but all had predicted target sites on the *TGFBR2 *UTR and could cause repression. miR-34b, which does not have a predicted target site, was used as a negative control. **(B) **Increase in *TGFBR2 *mRNA levels (measured by quantitative PCR) as the result of selective inhibition of expressed miRNAs. miRNA inhibitors (such as miR-20ai against miR-20a) were co-transfected with lacZ siRNA. Error bars = SD for three experiments. The result for miR-423 was not consistent with target prediction.

Two of the miRNAs predicted to have target sites in these regions are miR-20a (which has been shown to target *TGFBR2 *in megakaryocytes [49]) and miR-34a. Both these miRNAs were also expressed in the HaCaT cells used in this study (tested by microarray and Taqman qPCR). The fact that *TGFBR2 *is expressed in HaCaT cells despite the presence of these mRNAs raises the question of the strength of regulation of *TGFBR2 *expression, if any, by these miRNAs in this cell line. In transfection experiments using miRNA mimics and inhibitors, we found that *TGFBR2 *is indeed regulated by miR-20a and miR-34a in HaCaT cells. Luciferase activity of a construct containing the *TGFBR2 *3' UTR luciferase transcript and *TGFBR2 *mRNA levels were significantly reduced by transfected miR-20a or miR-34a (Figure 10A), and transfection of miR-20a or miR-34a inhibitors caused an increase in the *TGFBR2 *mRNA level (Figure 10B).

These examples suggest that the *TGFBR2 *mRNA is under the control of expressed miRNAs, and that changes in the expression levels of these miRNAs can modulate the expression level of the receptor. Inhibition of miR-20a or miR-34a leads to increased levels of *TGFBR2 *mRNA, whereas addition of exogenous miR-20a or miR34a leads to drastically reduced levels of *TGFBR2 *mRNA.

To probe the susceptibility of *TGFBR2 *to repression by non-expressed miRNAs, we tested two mimics of miRNAs not expressed in HaCaT cells: miR-373, which is predicted to target *TGFBR2*, and miR-34b, which is predicted not to target *TGFBR2*. Transfection of miR-373 did cause a decrease in *TGFBR2 *mRNA levels (Figure 10A), but consistent with target prediction, we did not observe an effect for miR-34b. Furthermore, we tested two more inhibitors of miRNAs expressed in HaCaT cells that are predicted to target *TGFBR2*: miR-93 and miR-423 (miR-20a and miR-93 have the same seed sequence). The result of the miR-423 assay was not consistent with expectation, but transfection of the miR-93 inhibitor resulted in increased *TGFBR2 *mRNA levels relative to a LacZ siRNA control (Figure 10B).

In summary, we found evidence of targeting of TGFBR2 by expressed miRNAs that was consistent with the siRNA off-target observations, with some evidence for regional selectivity, that is, regions of the *TGFBR2 *UTR being particularly sensitive to perturbation by small RNAs.

## Discussion

### TGF-β receptors are sensitive to silencing by RNAi

We performed a 6,000-gene siRNA screen for modulators of the TGF-β signal-transduction pathway. Although the GFP-SMAD2 translocation screen was indeed effective at isolating modulators of nuclear localization upon stimulus, it did not succeed in identifying novel components or modulators of the TGF-β pathway. All screen hits were found to be off-target effects against *TGFBR1 *and *TGFBR2 (*note that siRNAs intended to target *TGFBR1 *and *TGFBR2 *were not included in the screen, but used as controls), suggesting that there may be no other non-redundant components of the SMAD signaling pathway. However, a screen using a library that covers the entire genome would have to be performed to confirm this theory. Although the siRNA library was designed to target most known cancer and cancer-related genes (see Methods), other lesser-known genes that were not included in this screen may still be involved in TGF-β signaling.

The off-target hits were the most rate-limiting components of the TGF-β pathway, the type I and type II receptors. Our results recapitulate the outcomes of experiments conducted more than two decades ago, when Massagué *et al. *used an ethyl methanesulfonate mutagenesis approach to isolate TGF-β-resistant Mv1Lu mink lung epithelial cells on the basis of growth resistance to the cytostatic action of TGF-β after mutagenesis. Analysis of a total of 71 isolated clones showed that all had mutations that affected either the expression or protein sequence of TGFBR1 or TGFBR2 [50-55].

However, whereas mutagenesis and selection did not produce an observable bias towards TGFBR2 compared with TGFBR1 mutations in the mutagenesis study, *TGFBR2 *was the more frequent target for off-target effects in our screen, by a factor of five (Table 1). Rather than indicating a more rate-limiting role for TGFBR2 in the TGF-β pathway, the bias of these observed off-target effects against *TGFBR2 *can be interpreted to be due to the RNAi assay system itself. We considered the possibility that the observed bias was indicative of increased susceptibility of *TGFBR2 *mRNA to siRNA silencing compared with *TGFBR1*; however, even though we attempted to address this question, we did not identify any significant differences between *TGFBR1 *and *TGFBR2 *mRNA in terms of half-life or abundance (data not shown). In terms of increased potential for off-target effects, the 3' UTR of *TGFBR1 *might even be considered a better target, because it is twice as long (almost 5 kb) as the 3' UTR of *TGFBR2*.

Interestingly, many siRNAs presented seed matches to both receptor mRNAs, and resulted in a reduction in mRNA levels of both receptors. It is unlikely that the effect of the target genes of these siRNAs on the TGF-β pathway components is due to upstream regulatory effects on receptor transcription, as no hit genes were represented more than once, and several had no evidence of expression in the assay cell type (HaCaT). It is possible that the effects on the TGF-β receptor mRNAs are due to off-target effects on upstream regulators of receptor transcription, but such a gene was not identified by a global computational analysis of all hit siRNA sequences and 3' UTRs of the expressed genome. The transcriptional regulation of the receptors has been reported to be dependent on a number of transcription factors and signal-transduction pathways [56-60], which complicates this question.

The high rate of off-target effects against the TGF-β receptors in our screen suggests an increased sensitivity of the receptors to targeting by RNAi, which raises the possibility that they are important *in vivo *miRNA targets for modulation of cellular sensitivity and response to TGF-β. *TGFBR2 *and other genes implicated in neoplasia may be targets for regulation by aberrant miRNAs in cancer [49]. Dysregulation of involved miRNAs can lead to significant downregulation of the receptors, and can therefore counteract the growth-suppressing effect of TGF-β. In addition, as responsiveness to TGF-β is often lost by cells undergoing neoplastic transformation, manipulation of such an *in vivo *mechanism for TGF-β receptor regulation may be exploited by cells evading TGF-β cytostatic control or differentiation [24]. For example, *TGFBR2 *is potentially suppressed by aberrant expression of miR-20a in cancer [49] and by induction of miR-21 during adipogenic differentiation [61]. There are a number of other miRNAs predicted to target the 3' UTR regions of the type I and type II TGF-β receptors that are expressed in cancer cell lines [62], including miR-373, which is predicted to target *TGFBR2 *and has been implicated as an oncogene in testicular germ-cell tumors [63].

We found that the mRNA level of *TGFBR2 *is modulated by endogenous miRNAs in HaCaT cells. Inhibition, using an anti-sense inhibitor, of miR-20a or miR-34a, which are both expressed in HaCaT cells, caused an increase in *TGFBR2 *mRNA level, whereas transfection of miRNA mimics of these siRNAs caused a reduction.

It is possible that endogenous miRNAs act synergistically with the active siRNAs identified in our screen. As miRNAs have been shown to behave cooperatively in their silencing of endogenous transcripts via the 3' UTR, there is a possibility that selected siRNA hits mediate off-target effects by acting cooperatively with endogenous miRNAs regulating *TGFBR2 *transcripts [64,65]. The siRNA target regions could indicate proximal miRNA target areas in the *TGFBR2 *mRNA, as has recently been suggested [18]. Based on our results, we speculate that the susceptibility of the receptor mRNA, especially that of *TGFBR2*, is due to cooperativity with an existing *in vivo *mechanism for *TGFBR2 *receptor regulation through RNAi. The TGF-β receptor transcripts may be in a 'primed' RNAi state due to endogenous miRNA regulation and RISC association, resulting in constant association with the RISC.

Cooperativity between endogenous miRNAs and siRNAs may also be an explanation for the higher than expected number of siRNA hits with silencing activities against both *TGFBR1 *and *TGFBR2 *(based on the number of siRNAs targeting *TGFBR1 *or *TGFBR2 *individually, the expected number of siRNAs targeting both receptors is 2 × 10^-5^, but the observed number was 2 × 10^-3^). As seen in our siRNA hit list, silencing of only one of the receptors was sufficient to significantly affect TGF-β signaling, so selection for double-silencers cannot be used to explain the results. Certain endogenous miRNAs could be involved in the regulation of both TGF-β receptors, and the introduction of siRNAs with binding sites within an optimal distance from the miRNA target sites could lead to cooperative activity between siRNAs and miRNAs. Owing to the very large number of possible combinations, we were unable to test this issue experimentally. Combining improved target prediction and extensive testing of siRNAs and of miRNA inhibitors may eventually answer this question.

### Mechanisms of RNAi off-target effects

Although some degree of off-target effects in our screen had been anticipated, the magnitude and specificity of off-target effects found was unexpected. We analyzed 176 individual siRNA sequences *in vitro *for insight into the sequence basis of off-target effects against the *TGFBR1 *and *TGFBR2 *mRNAs. Most of the off-target effects appeared to be mediated by siRNAs containing seed matches, predominantly in the guide strand, to conserved regions of the 3' UTR of the mRNAs transcripts.

Our analysis identified a number of characteristics that appear to be similar to those described for miRNA targeting. The siRNA hit population displayed significant enrichment for miRNA-like traits, such as a heptamer seed match, t1A anchors, t9W, G:U wobbles in the seed region, 3' sequence matches, and positional bias along the 3' UTR regions and conserved regions. One parameter that emerged as a result of our analysis was a match at position 16 of the siRNA, which seemed to be one of the most highly enriched among the siRNA hits, both in our dataset and in siRNA datasets reported previously [20,22].

The importance of position 16 for siRNA targeting has been noted previously [22], but has not yet been described in the context of siRNA off-target effects. Recent structural work revealed that the mechanism of siRNA target binding most likely involves the release of the 3' end of the siRNA from the Paz domain [66]. In this way, the 5' end of the siRNA remains tethered inside the mid domain of the Argonaute protein, and there is a helical conformation of positions 12-15, allowing catalytic residues in Argonaute to be placed optimally in relation to the target mRNA. Detailed truncation experiments showed that the release of the 3' end of the guide siRNA from the PAZ domain is driven by residue matching up to position 16 [66]. Interestingly, this release from the PAZ domain confirmed earlier biochemical work by the Zamore group, who first suggested the 'two-state model' [67]. Other systematic analyses have also shown a role for position 16 in the efficacy of siRNA [68], and position 16 was one of only five positions at which mutations showed effective discrimination against the wild-type SOD1 reporter [22].

We also observed an enrichment of seed matches containing G:U wobbles. Surprisingly, these G:U wobble pairs occurred preferentially at certain positions of the siRNA, with a preference for a G at siRNA positions 3 and 8, and U at positions 2 and 4.

In our study, we also found a number of siRNA hits that had no seed matches to either the 3' UTR or the ORF regions of the receptors. When we tested the siRNA hits without seed matches to the 3' UTR of the receptors, we found they also resulted in a reduction in mRNA levels of the TGF-β receptors and in silencing against the 3' UTR regions in a luciferase assay.

All our data indicate the importance of a perfect seed match, but although a seed match is a strong predictor for siRNA silencing, it is by no means essential. Existing seed-match based miRNA target prediction methods, such as TargetScan, have included parameters in addition to the seed, such as binding of the 3' end of the miRNA, local A:U content, target structure and conservation, all of which result in increased prediction specificity. However, each increase in specificity is accompanied by a significant decrease in sensitivity. A balanced weighting of these features can help, but ultimately, the strict requirement of a seed match limits this method. Each additional component of the miRNA mechanism identified should allow relaxation of the strict requirement for a seed match. For example, miRNA-target relationships with mismatches and G:U wobbles in the seed region have been identified, and it has been hypothesized that a significant part of this specificity may depend on the mRNA sequence or secondary structure. In addition, the number of siRNA target sites in a particular sequence can also increase silencing efficacy.

One striking observation in our analysis is that existing seed-based methods drastically overpredict the number of siRNA target sites. In our example, only 61 of >3000 siRNA sequences with at least one heptamer seed match against the 3' UTR of *TGFBR2 *had the ability to downregulate *TGFBR2*.

### Improving future RNAi screens

The extent of the off-target effects seen in our study may be specific to the assay and siRNA library we used. However, our results do suggest that some caution should be taken in interpreting results from siRNA screens. We join other groups who call for some vigilance when using large- or small-scale RNAi techniques [23,69]. A strict requirement for multiple potent siRNAs against one gene may reduce the likelihood of overinterpreting screen hits resulting from off-target effects [70]. In addition, any presumed biological effect due to target-specific knockdown ideally should be verified by observing its reversal after reintroducing the particular gene product in knockdown experiments.

There is an ongoing need for further improvement in siRNA design. Our results suggest that avoiding G and U nucleotides in the seed region during siRNA design could reduce off-target effects to some extent, and there may be other position-specific preferences that could be exploited. Chemical modification of certain siRNA positions has recently been shown to reduce off-target effects [71]. Other RNAi methods with less potential for off-target effects, such as endoribonuclease-prepared siRNAs, may be more appropriate for large-scale screens of signal-transduction pathways [72], although they present novel challenges, such as the potential induction of interferon response in certain cell types [73].

Furthermore, saturation of the miRNA machinery by transfection of siRNAs and the subsequent release of miRNA inhibition of other endogenous genes can have unintended consequences [74], but simply lowering siRNA concentrations will compromise targeting efficiency. mRNA-specific features such as mRNA half-life should also be taken into account [75]. If all these challenges can be overcome, miRNAs, siRNAs or their analogues may have even greater potential in therapeutics.

## Methods

### siRNA library

The siRNA library used (synthesized at Memorial Sloan-Kettering Cancer Center (MSKCC) by the Organic Synthesis Core Facility) encompasses ~21,000 RNA duplexes targeting approximately 6,000 human genes, and each gene is targeted by an average of three independent siRNAs (see Additional File 2 Table S1). The library was designed to cover most known genes or genes with suspected function, with a bias toward cancer biology, including all genes reported to be causally related or even correlated with cancer *in vivo *or transformation *in vitro*. In addition, all known kinases, phosphatases, cell-surface receptors and signal-transduction components were represented.

Library design was based on rules by Reynolds *et al.*[36] and were further refined through quantitative analysis of suppression of two test proteins (Lack and Rab-6), using an extensive array of sequences (more than 500 individual siRNAs) derived from these genes [17]. These newly derived rules represent a compromise between siRNA efficacy (0.74 probability of >70% knockdown of protein per siRNA, and a 0.98 probability that one out of three siRNAs will work) and the ability to find such a sequence in a particular gene. In those few cases where a sequence could not be found, the rules were relaxed, with a reduction in probability (for three siRNAs) to 0.91. The design rules for the siRNAs were as follows (defined for the siRNA guide strand): 1) A or U at position 1 and 9, and/or G or C at position 19 (the position 1 A or U has priority); 2) three or more A or U nucleotides at positions 1 to 7; 3) no single-nucleotide polymorphisms; 4) location at least 100 nucleotides from a start or stop codon; 5) uniqueness of the sequence verified by BLAST analysis; and 6) selection of shared sequences for genes with multiple transcripts. A summary of the library composition is included (see Additional File 3 Table S7). All sequences were 19-mers with an additional dTdT 3' overhang. All RNA molecules were purified by high-performance liquid chromatography.

### Screen

The screen was performed (MSKCC High-Throughput Screening Facility) in 384-well plates with a plating density of 3,000 cells per well. Cells were transfected with siRNA the day after plating, and then stimulated with 100 pmol/l TGF-β-1 48 hours after transfection. Cells were stained with Hoechst stain, and fixed 90 minutes after stimulation.

Confocal images (one frame per well) were acquired (IN Cell Analyzer 3000; GE Healthcare, Waukesha, WI, USA). Nuclear translocation data were extracted using the nuclear-trafficking analysis module, and cell size and GFP intensity ranges were selected to achieve maximum separation between positive and negative controls, resulting in an average *Z*' score of 0.5. Images of all screen hits were inspected visually, and hits resulting from poorly aligned images (GFP versus nuclear-staining channel) or poor cell morphology were flagged and excluded from downstream analyses. Selected representative images are available at http://cbio.mskcc.org/tgf-beta_screen/.

### Normalization of screen results

Initial analysis of the raw data revealed experimental bias, as elevated N:C ratios were found along the edges of all plates relative to the ratios in control wells located in the middle of each plate. Further analysis showed that this effect was dependent on cell density; that is, N:C ratios were inversely correlated with the number of cells imaged per well (see Additional File 1 Figure S1). The lower cell density in the outside wells was most likely due to uneven settling of the cells, as cells tend to migrate to warmer temperatures while they are settling [76], which would lead to an increased cell density towards the outer edges of the outside wells and a decrease in the center of the wells, which was the area from which all our images were acquired. To correct for this effect, we developed a linear regression method to normalize N:C ratios by the cell number assayed per well.

First, individual plate baseline and variability effects were corrected for by standardizing each plate to a mean of 0 and SD of 1. To avoid confounding effects, where N:C outliers were generated by aberrant cell count or plate position, N:C ratios were corrected with respect to both cell count and plate position. The correction was performed in Matlab as follows (code available on request). For each well, a linear bias function was estimated, which describes how cell count affects the N:C ratio in that region of the plate, using the linear equation

where N is the cell count, and A and B are coefficients. The coefficients A and B were fitted using linear least squares, in a robust procedure that took into account information from neighboring wells (nearest neighbor kernel estimation). Wells with measurements for <400 cells were removed from the analysis. Having estimated A and B, corrected N:C ratios were obtained by subtracting the bias term. In a final correction step, corrected N:C values were standardized to σ = 1 across the 65 plates.

### Cell lines and transfection

HaCaT keratinocytes were maintained in Dulbecco's modified Eagle's medium supplemented with 10% fetal bovine serum. The cell-culture medium also contained 100 U/ml penicillin and 100g/ml streptomycin, 2 mmol/l L-glutamine and 1 ug/ml fungizone. Transient transfection of siRNA ribonucleotide oligomers and siRNA/DNA co-transfections were performed using a commercial reagent (Lipofectamine 2000, Invitrogen, Carlsbad, CA, USA), in accordance with the manufacturer's instructions, as was transfection of DNA into HaCaT cells (Lipofectamine, Invitrogen).

### Generation of the GFP-SMAD2 cell line

The coding sequence for SMAD2 was amplified by PCR from human cDNA and inserted into a cloning vector (Zero Blunt 2.0, Invitrogen). Enhanced (E)GFP was amplified by PCR from an EGFP plasmid (Clontech, Mountain View, CA, USA), using modified primers that contained a *Kpn*I and a *XhoI *restriction site in the 5' region of the forward primer, and a *Kpn*I site in the 5' region of the reverse primer. EGFP was subsequently cloned into the *Kpn*I site of the SMAD2 Zero Blunt plasmid, and the EGFP-SMAD2 fusion sequence was then cloned into the *Xho*I site of the mammalian expression plasmid pCAGGS.

To generate the stable EGFP-SMAD2 cell line, the EGFP-SMAD2 expression plasmid was transfected into HaCaT keratinocytes. After 2 weeks of culture, cells were subjected to fluorescence-activated cell sorting (FACS) to isolate stably transfected EGFP-positive cells (~0.5%). These cells were then grown up and subjected to another round of sorting, in which only cells with moderate EGFP levels were selected.

### Generation of luciferase reporter constructs

The coding sequence for firefly luciferase was cloned into the *Kpn*I and *Mlu*I sites of the mammalian expression vector pCMV5 to generate constitutive luciferase expression. The PCR-amplified 3' UTR (NM_001024847, nucleotides 2108-4680) or ORF (NM_001024847, nucleotides 383-2161) regions of TGFBR2 were inserted immediately downstream of the firefly luciferase stop codon.

### Luciferase assays

Luciferase assays in SW13 cells with a mammalian TGF-β-inducible luciferase reporter construct (SBE-4X) [77] were performed as described previously [78]. A constitutively active cytomegalovirus-*Renilla *luciferase plasmid (Promega, Madison, WI, USA) was included as an internal control. For 3' UTR and control reporter assays, 50 ng each of reporter plasmid were transfected with 60 pmol siRNA per well in 12-well plates, with each transfection performed in triplicate. Luciferase activity in these assays was evaluated 24 hours after transfection.

### miRNA mimics and miRNA inhibitors

miRNA mimic duplexes were synthesized and annealed (Sigma Proligo, St. Louis, MO, USA) with the sequences shown in Table 2 (from Sanger miRBase release 9.2). miRNA inhibitors were obtained from a commercial source (anti-miR; (Ambion, Austin, TX, USA). miRNA mimic duplexes and miRNA inhibitors were transfected using the same protocol and amounts as the siRNA duplexes described above.

**Table 2:** Sequences used for generation of microRNAs.

miR-20a	UAAAGUGCUUAUAGUGCAGGUAG
miR-20a*	ACUGCAUUAUGAGCACUUAAAGU

miR-34b	UAGGCAGUGUCAUUAGCUGAUUG

miR-34b*	AUCACUAACUCCACUGCCAUCA

miR-373	GAAGUGCUUCGAUUUUGGGGUGU

miR-373*	ACUCAAAAUGGGGGCGCUUUCC

### Antibodies and reagents

We used antibodies recognizing phospho-tail SMAD2 Ser-465/467 (Cell Signaling, Danvers, MA, USA) and murine antibodies against α-tubulin (Sigma-Aldrich, St. Louis, MO, USA). The SMAD2 antibody (183 to 273 peptides) has been described previously [79]. For immunofluorescence experiments, HaCaT cells were plated on chamber slides (LabTek II; Nunc, Rochester, NY, USA) and treated with 100 pmol/l TGF-β-1 for 60 minutes. Cells were fixed in 4% paraformaldehyde. Alexa Fluor 488-conjugated goat anti-rabbit IgG (Invitrogen) was used as a secondary antibody.

### Quantification of mRNA by real-time PCR analysis

Total RNA from HaCaT cells was harvested (RNeasy Kit; Qiagen, Valencia, CA, USA). cDNA was synthesized from 100 ng of purified RNA (High-Capacity cDNA Archive Kit for RT-PCR; Applied Biosystems, Foster City, CA, USA) in accordance with the manufacturer's protocol. Quantitative reverse transcriptase PCR was performed in an automated PCR system (7900HT; Applied Biosystems). All reactions were performed in a volume of 10 μl containing cDNA template equivalent to 500 pg of RNA template, 0.1 μmol/l primers (Table 3) and 5 μl of 2× SYBR Green I Master Mix (Applied Biosystems). Each sample was analyzed in quadruplicate. PCR cycling parameters were: 50°C for 2 minutes and 95°C for 10 minutes, then 40 cycles of 94°C for 15 seconds and 60°C for 1 minute. Data analysis was performed using the comparative Ct method (SDS, version 2.2.2; Applied Biosystems). Hypoxanthine phosphoribosyltransferase (HPRT) 1 and SMAD4 were used for normalization.

**Table 3:** Primers used for quantitative PCR analysis.

CDKN1A-fw	CCGAGGCACTCAGAGGAG
CDKN1A-rev	AGCTGCTCGCTGTCCACT

SERPINE1-fw	AAGGCACCTCTGAGAACTTCA

SERPINE1-rev	CCCAGGACTAGGCAGGTG

HPRT1-fw	TGACCTTGATTTATTTTGCATACC

HPRT1-rev	CGAGCAAGACGTTCAGTCCT

SMAD4-fw	CCTGTTCACAATGAGCTTGC

SMAD4-rev	GCAATGGAACACCAATACTCAG

SMAD7-fw	AGGGGGAACGAATTATCTGG

SMAD7-rev	ACCACGCACCAGTGTGAC

TGFBR1-fw	TGTTACGTCATGAAAACATCCTG

TGFBR1-rev	ACCAGAGCTGAGTCCAAGTACC

TGFBR2-fw	GACCAGAAATTCCCAGCTTCT

TGFBR2-rev	CAACGTCTCACACACCATCTG

### Quantification of mRNA by branched DNA assay

HaCaT cells were transiently transfected with siRNA in 12-well plates. Cells were harvested 48 hours after transfection and processed (QuantiGene Reagent System; Panomics, Fremont, CA, USA) in accordance with manufacturer's instructions. Cells were lysed in a volume of 1.5 ml, and 30 ml of lysate probed in duplicate with the following QuantiGene probe sets: human TGFBR1-PA-10418-02, human TGFBR2-PA-10587-02 and human HPRT1-PA-10389-02 (used for normalization).

### Quantification of miRNAs by real-time PCR analysis and by microarray

RNA was isolated (mirVana™ miRNA Isolation Kit; Ambion). Quantitative PCR analysis of selected miRNAs in HaCaT cells was performed (TaqMan miRNA Assays; Applied Biosystems) in accordance with the manufacturer's instructions. In parallel, miRNA profiles of HaCaT cells were generated (Human miRNA Microarray Kit, version 1; Agilent, Santa Clara, CA, USA).

### 3' UTR sequence analysis

RefSeq sequences NM_001130916 and NM_001024847 were used for sequence analysis of TGFBR1 and TGFBR2, respectively. Ensembl version 58 (143,127 3' UTRs) was used for the identification of the most frequent off-target genes.

## Competing interests

The authors declare that they have no competing interests.

## Authors' contributions

NS, DRM, DSM and CS wrote the manuscript. NS and DDA designed the assay and adapted it for screening. DDA performed the high-throughput screen. NS, DDA and SN analyzed the screening results. DRM and NS performed the experimental hit validation. NS, AJ, DSM, WW and SN analyzed the sequence determinants of the off-target effects. CS and JM conceived and guided the project. All authors read and approved the final manuscript.

## Supplementary Material

Additional file 1 **Supplementary Figures**. **Figure S1. Effect of cell number on nuclear:cytosolic (N:C) ratio and normalization**. Correlation between cell number and N:C ratio (left) before and (right) after normalization. **Figure S2. Chemical inhibitors against protein kinase A and A-kinase anchor protein (AKAP), two candidate hits in this screen, had no effect on transforming growth factor (TGF)-β-induced expression of *SMAD7***. Two inhibitors of protein kinase A (14-22 and H89), an inhibitor against AKAP (Ht31) and a *TGFBR2 *inhibitor (SB431542) were tested on HaCaT cells exposed to different concentrations of TGF-β. *SMAD7 *induction was inhibited only in the presence of the *TGFBR2 *inhibitor. Error bars on each column are the mean ± SD of three experiments. **Figure S3. Small interfering (si)RNA hits but not other siRNAs designed for the same target silenced a TGFBR2 **3' **UTR luciferase construct**. siRNAs designed to target the same genes as those identified as hits in the screen were tested for their effect in silencing a TGFBR2 3' UTR luciferase construct. The screen hit caused significant silencing of the luciferase, whereas the other matched siRNA did not. The control siRNA for A-kinase anchor protein (AKAP) P13 (Ambion) was not used in the screen. The data are presented (*y*-axis) as the relative repression of firefly luciferase expression standardized to *Renilla *luciferase as a transfection control. Integers (for example 9, 18) label ranked siRNA hits. Error bars on each column are the mean ± SD of three experiments. **Figure S4. Screen hits with a verified effect on *TGFBR2 *were enriched for small interfering (si)RNA seed matches to the *TGFBR2 *ORF**. Screen hits with measured effects on *TGFBR2 *(*n *= 106) were found to be slightly enriched for heptamer seed matches to the ORF of TGFBR2 compared with the non-hit control group (*r *= 1.37). This enrichment was significantly higher after removing all siRNA sequences with one or more heptamer seed matches to the TGFBR2 3' UTR (*r *= 2.03, *P *= 0.0046, Fisher's exact test). Click here for file

Additional file 2 **Supplementary Tables 1-3**. Table S1: Full screen results, Table S2: Target gene analysis (siRNA knockdown efficiencies), Table S3: Detailed analysis of 193 screen hits Click here for file

Additional file 3 **Supplementary Tables 4-7**. Table S4: *TGFBR2* is the most significantly off-targeted gene (guide strand). Table S5: *TGFBR2* is among the most off-targeted gene (passenger strand). Table S6: *TGFBR2* is the only significantly off-targeted gene (both strands). Table S7: siRNA library composition (guide strand). Click here for file
