# Supplementary material for: Off-target effects dominate a large-scale RNAi screen for modulators of the TGF-β pathway and reveal microRNA regulation of TGFBR2
Source: Silence. 2011 Mar 14;2:3. doi: 10.1186/1758-907X-2-3 (PMC3068080; doi:10.1186/1758-907X-2-3)

Figure S1. Effect of cell number on N/C ratio and normalization.

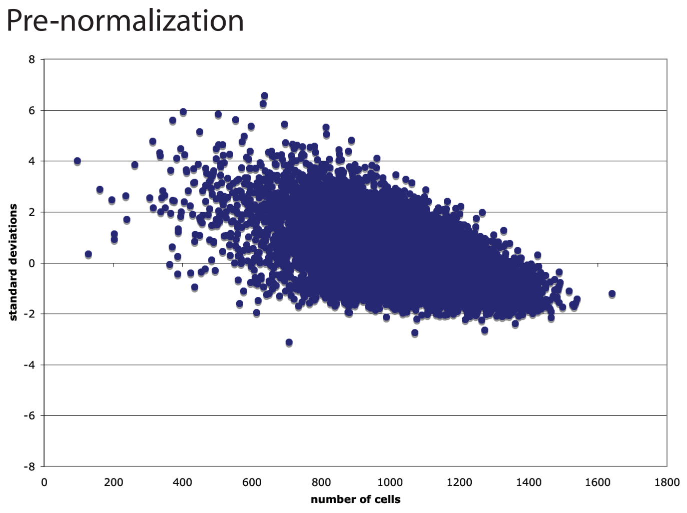

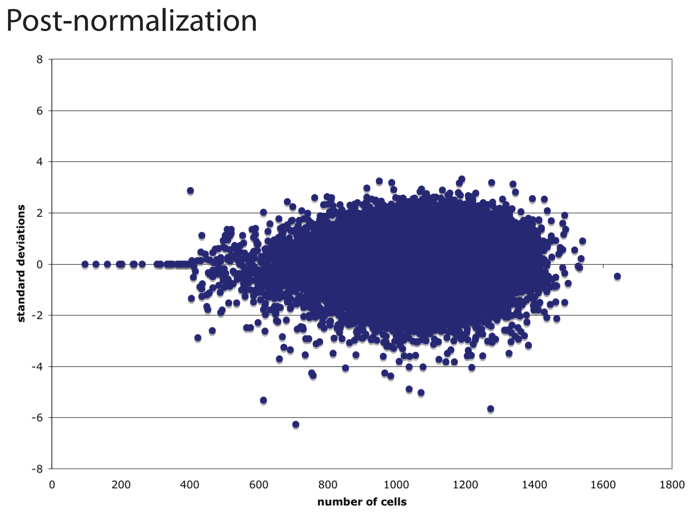

Figure S2. Chemical inhibitors against Protein Kinase A and AKAP, two candidate hits in this screen, have no effect on TGFβ-induced expression of SMAD7.

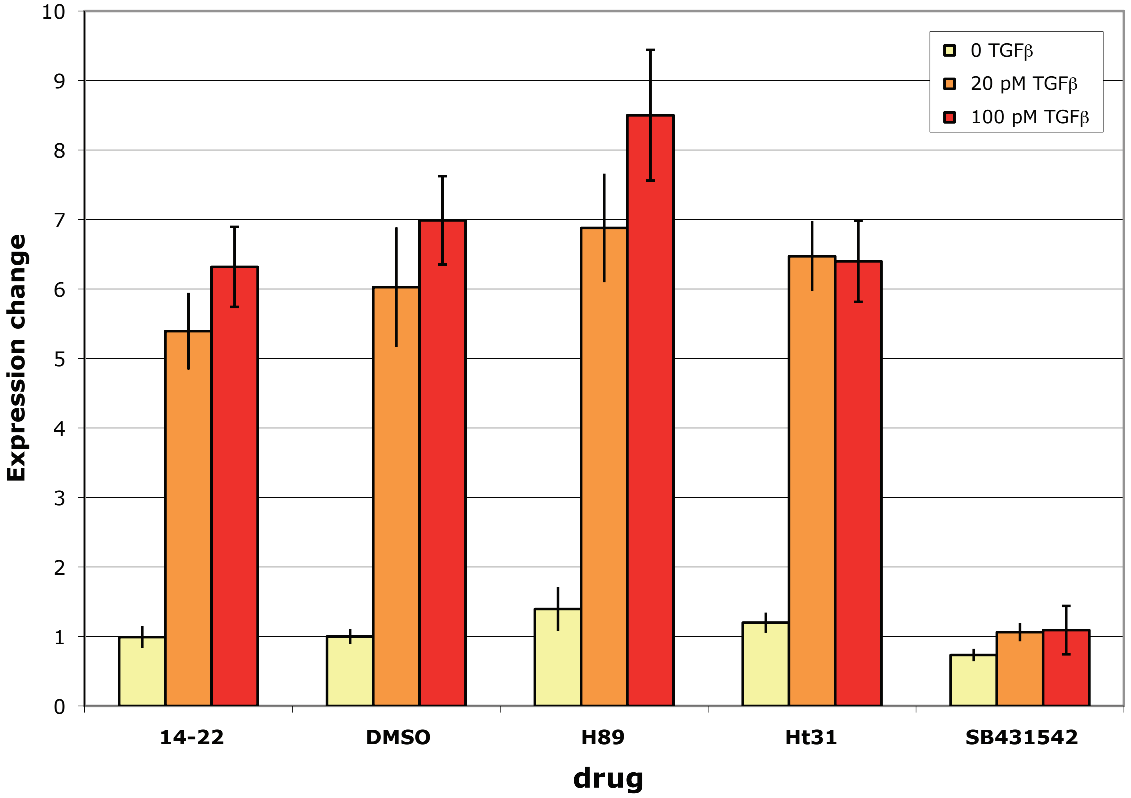

Figure S3. Hit siRNAs but not other siRNAs designed for the same target silence a TGFBR2 3'UTR Luciferase construct.

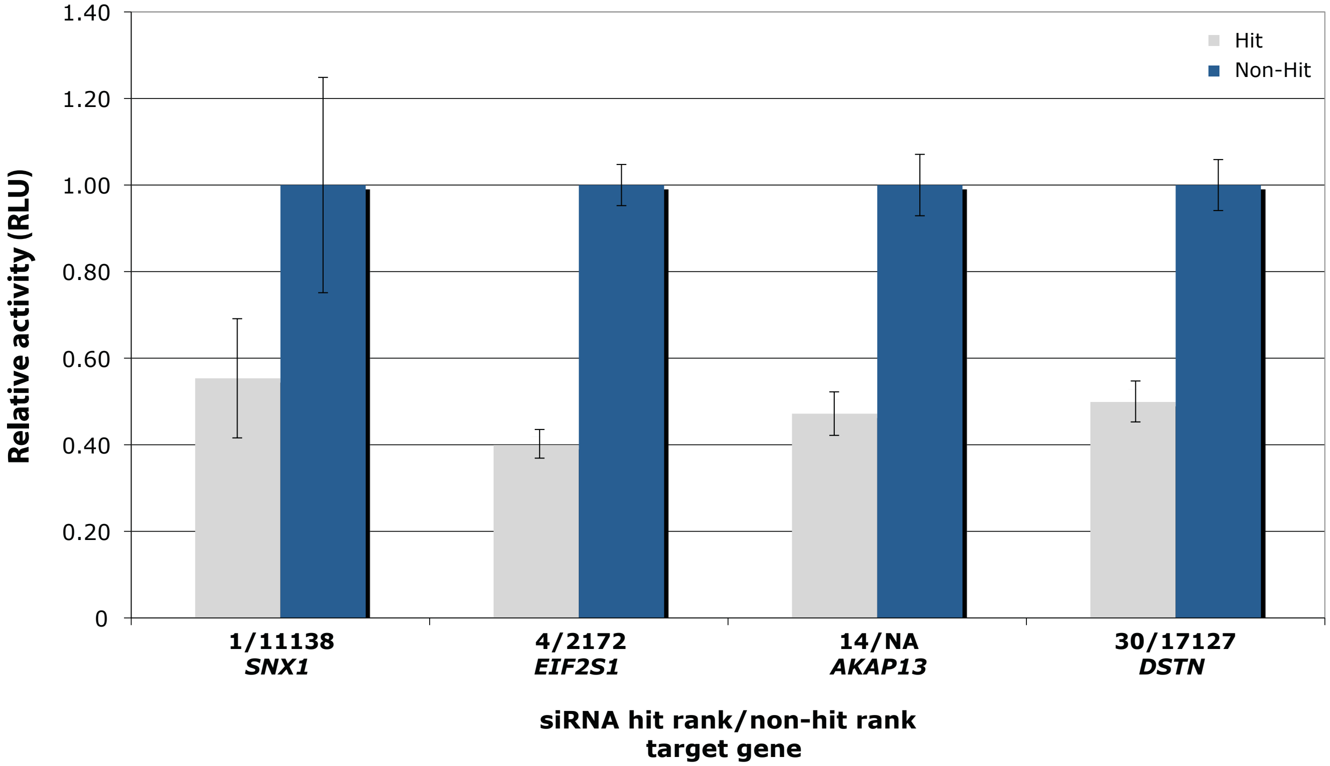

Figure S4. Screen hits with a verified effect on TGFBR2 are enriched for siRNA seed matches to the TGFBR2 ORF.

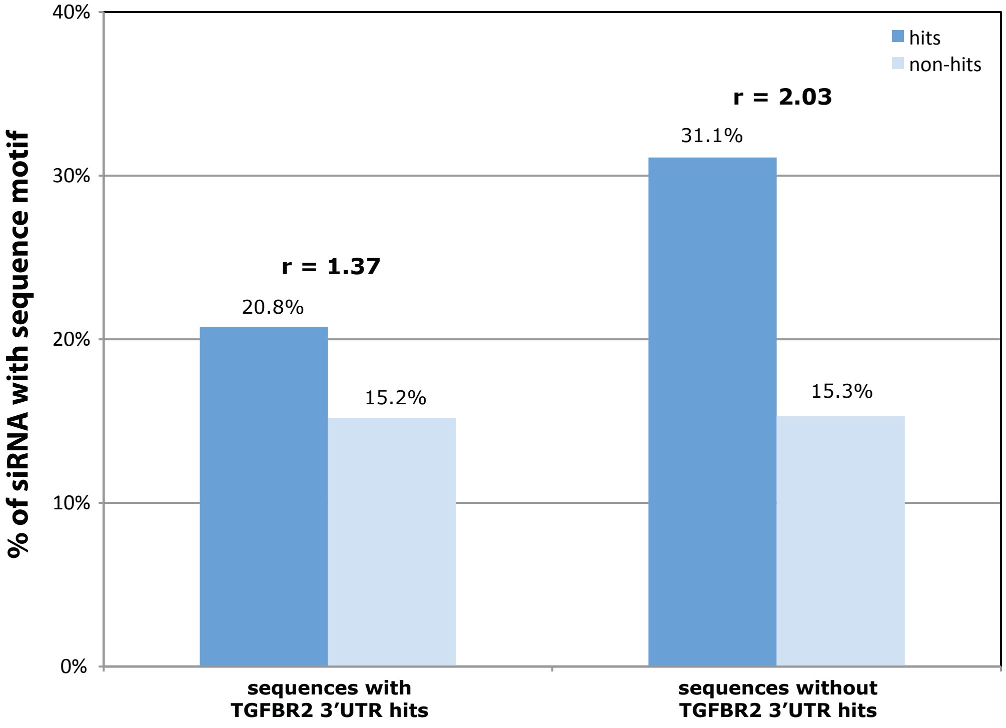

Supplement: Additional file 1 — Supplementary Figures. Figure S1. Effect of cell number on nuclear:cytosolic (N:C) ratio and normalization. Correlation between cell number and N:C ratio (left) before and (right) after normalization. Figure S2. Chemical inhibitors against protein kinase A and A-kinase anchor protein (AKAP), two candidate hits in this screen, had no effect on transforming growth factor (TGF)-β-induced expression of SMAD7. Two inhibitors of protein kinase A (14-22 and H89), an inhibitor against AKAP (Ht31) and a TGFBR2 inhibitor (SB431542) were tested on HaCaT cells exposed to different concentrations of TGF-β. SMAD7 induction was inhibited only in the presence of the TGFBR2 inhibitor. Error bars on each column are the mean ± SD of three experiments. Figure S3. Small interfering (si)RNA hits but not other siRNAs designed for the same target silenced a TGFBR2 3' UTR luciferase construct. siRNAs designed to target the same genes as those identified as hits in the screen were tested for their effect in silencing a TGFBR2 3' UTR luciferase construct. The screen hit caused significant silencing of the luciferase, whereas the other matched siRNA did not. The control siRNA for A-kinase anchor protein (AKAP) P13 (Ambion) was not used in the screen. The data are presented (y-axis) as the relative repression of firefly luciferase expression standardized to Renilla luciferase as a transfection control. Integers (for example 9, 18) label ranked siRNA hits. Error bars on each column are the mean ± SD of three experiments. Figure S4. Screen hits with a verified effect on TGFBR2 were enriched for small interfering (si)RNA seed matches to the TGFBR2 ORF. Screen hits with measured effects on TGFBR2 (n = 106) were found to be slightly enriched for heptamer seed matches to the ORF of TGFBR2 compared with the non-hit control group (r = 1.37). This enrichment was significantly higher after removing all siRNA sequences with one or more heptamer seed matches to the TGFBR2 3' UTR (r = 2.03, P = 0.0046, Fish [file 1758-907X-2-3-S1.PDF]
