# Supplementary material for: Off-target effects dominate a large-scale RNAi screen for modulators of the TGF-β pathway and reveal microRNA regulation of TGFBR2
Source: Silence. 2011 Mar 14;2:3. doi: 10.1186/1758-907X-2-3 (PMC3068080; doi:10.1186/1758-907X-2-3)
Supplement: Additional file 3 — Supplementary Tables 4-7. Table S4: TGFBR2 is the most significantly off-targeted gene (guide strand). Table S5: TGFBR2 is among the most off-targeted gene (passenger strand). Table S6: TGFBR2 is the only significantly off-targeted gene (both strands). Table S7: siRNA library composition (guide strand). [file 1758-907X-2-3-S3.PDF]

Additional File 3

Tables S1-3 are combined in **Additional File 2** (XLS format):

Table S1. Full screen results

Table S2. Target gene analysis (siRNA knockdown efficiencies)

Table S3. Detailed analysis of 193 screen hits

Table S4. ***TGFBR2*** **is the most significantly off-targeted gene (guide strand).**

**rank**	**gene**	**UTR length**	**count in hits**	**count in control**	**enrichment**	**p-value**	**Ensembl ID**
1	***TGFBR2***	2535	78	729	2.674897	**6.42E-12**	ENST00000295754
2	***CD248***	267	19	92	5.163043	**1.14E-03**	ENST00000311330
3	***C3orf52***	563	26	182	3.571429	**2.63E-03**	ENST00000480282
4	***GALE***	345	21	125	4.2	**4.82E-03**	ENST00000456977
5	***C9orf107***	201	16	71	5.633803	**5.31E-03**	ENST00000374801
6	***SBF1***	391	22	140	3.928571	**6.74E-03**	ENST00000356279
7	***TYMP***	500	24	173	3.468208	**1.44E-02**	ENST00000426603
8	***KCNC2***	1124	35	338	2.588757	**1.87E-02**	ENST00000298972
9	*CSN3*	201	14	68	5.147059	8.52E-02	ENST00000304954
10	*SYPL1*	505	21	152	3.453947	8.68E-02	ENST00000470347

The siRNA seed matches with 3'UTRs of all genes were compared between the top 200 and a control group of non-hits (screen ranks 12,001-17,000). The significance of enrichment was highest for TGFBR2 (Fisher's exact test).

Table S5. ***TGFBR2*** **is among the most off-targeted gene (passenger strand).**

**rank**	**gene**	**UTR length**	**count in hits**	**count in control**	**enrichment**	**p-value**	**Ensembl ID**
1	*C17orf95*	193	10	54	4.62963	5.53E+00	ENST00000317409
2	*MAP3K13*	220	11	74	3.716216	1.44E+01	ENST00000454237
3	*CDK15*	805	27	329	2.051672	1.79E+01	ENST00000451080
4	*ODZ2*	1347	30	389	1.928021	2.26E+01	ENST00000403607
5	*CETN1*	597	21	231	2.272727	2.38E+01	ENST00000327228
6	*TET3*	395	14	124	2.822581	3.04E+01	ENST00000409262
7	*TGFBR2*	2535	50	801	1.560549	3.70E+01	ENST00000295754
8	*CALHM2*	171	7	35	5	3.81E+01	ENST00000393235
9	*LY86*	354	15	147	2.55102	5.14E+01	ENST00000230568
10	*AURKA*	805	23	285	2.017544	5.98E+01	ENST00000395907

The siRNA seed matches with 3'UTRs of all genes were compared between the top 200 and a control group of non-hits (screen ranks 12,001-17,000). The significance of enrichment was highest for TGFBR2 (Fisher's exact test).

Table S6. ***TGFBR2*** **is the only significantly off-targeted gene (both strands).**

rank	gene	UTR length	count in hits	count in control	enrichment	p	Ensembl ID
1	***TGFBR2***	2535	128	1530	2.091503	**1.22E-11**	ENST00000295754
2	*CD248*	267	25	207	3.019324	1.20E-01	ENST00000311330
3	*C3orf52*	563	37	394	2.347716	1.45E-01	ENST00000480282
4	*TYMP*	500	35	377	2.320955	3.38E-01	ENST00000426603
5	*GALE*	345	29	288	2.517361	5.15E-01	ENST00000456977
6	*KCNC2*	1124	51	690	1.847826	1.24E+00	ENST00000298972
7	*KIF9*	255	25	241	2.593361	1.31E+00	ENST00000444589
8	*MAP3K13*	220	19	155	3.064516	1.55E+00	ENST00000454237
9	*PLEKHA6*	1567	76	1199	1.584654	2.06E+00	ENST00000272203
10	*SBF1*	391	27	283	2.385159	2.43E+00	ENST00000356279

The siRNA seed matches with 3'UTRs of all genes were compared between the top 200 and a control group of non-hits (screen ranks 12,001-17,000). The significance of enrichment was highest for TGFBR2 (Fisher's exact test).

Table S7. ***siRNA library composition (guide strand)*.**

	1	2	3	4	5	6	7	8	9	10	11	12	13	14	15	16	17	18	19
**A**	45.2%	18.6%	23.6%	24.7%	24.1%	22.9%	24.3%	23.2%	22.6%	26.0%	23.0%	23.0%	24.6%	24.0%	21.4%	22.7%	21.9%	21.8%	18.6%
**C**	2.9%	24.8%	25.7%	24.5%	24.6%	25.4%	24.5%	25.2%	26.8%	23.2%	25.4%	26.0%	24.7%	24.8%	27.1%	26.7%	26.6%	26.7%	31.5%
**G**	3.2%	30.1%	24.5%	23.2%	23.7%	25.5%	23.3%	24.6%	25.8%	22.7%	24.9%	25.6%	23.7%	24.3%	27.2%	26.1%	25.8%	26.4%	25.9%
**U**	48.7%	26.5%	26.2%	27.7%	27.6%	26.2%	27.9%	27.0%	24.9%	28.1%	26.8%	25.4%	27.0%	26.9%	24.4%	24.6%	25.7%	25.1%	24.0%

Average occurrence of the four nucleotides in each position of the siRNA guide strand. Positions 20 and 21 are dTdT overhangs in both strands.

Supplemental Web Material:

Selected, representative images at: http://cbio.mskcc.org/tgf-beta_screen/
